# Interpretable Machine Learning on Metabolomics Data Reveals Biomarkers for Parkinson’s Disease

**DOI:** 10.1021/acscentsci.2c01468

**Published:** 2023-05-09

**Authors:** J. Diana Zhang, Chonghua Xue, Vijaya B. Kolachalama, William A. Donald

**Affiliations:** † School of Chemistry, University of New South Wales, Sydney 2052, Australia; ‡ Department of Medicine, Boston University School of Medicine, Boston, Massachusetts 02118, United States; § Department of Computer Science and Faculty of Computing & Data Sciences, Boston University, Boston, Massachusetts 02215, United States

## Abstract

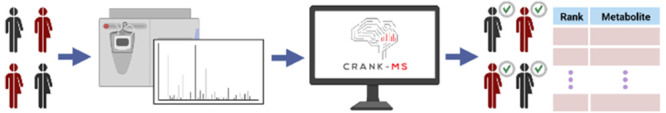

The use of machine learning (ML) with metabolomics provides opportunities for the early diagnosis of disease. However, the accuracy of ML and extent of information obtained from metabolomics can be limited owing to challenges associated with interpreting disease prediction models and analyzing many chemical features with abundances that are correlated and “noisy”. Here, we report an interpretable neural network (NN) framework to accurately predict disease and identify significant biomarkers using whole metabolomics data sets without *a priori* feature selection. The performance of the NN approach for predicting Parkinson’s disease (PD) from blood plasma metabolomics data is significantly higher than other ML methods with a mean area under the curve of >0.995. PD-specific markers that predate clinical PD diagnosis and contribute significantly to early disease prediction were identified including an exogenous polyfluoroalkyl substance. It is anticipated that this accurate and interpretable NN-based approach can improve diagnostic performance for many diseases using metabolomics and other untargeted ‘omics methods.

## Abstract 2

Interpretable machine learning on whole metabolomics data sets can significantly improve diagnostic performance and retrospectively “mine” disease biomarkers.

## Introduction

The rate of Parkinson’s disease (PD) is growing more rapidly than any other neurological disease.^1^ PD is typically diagnosed according to a clinical criteria of motor symptoms which include bradykinesia (slowness of movement), a resting tremor, and rigidity.^2^ However, the onset of atypical nonmotor symptoms such as sleep disorder, constipation, apathy, and loss of smell can predate clinically relevant symptoms by several years to decades.^3−5^ In addition, for patients who present with Parkinson-like symptoms, the current process for identifying PD can often be inconclusive. For example, according to a meta-analysis by Rizzo et al.,^6^ the overall diagnostic accuracy for PD based on an initial clinical assessment by movement disorder experts is 80%. Accurate identification of PD using biomarker signatures rather than relying primarily on physical symptoms would be highly beneficial.

Biomarkers associated with metabolic processes are used extensively for understanding, diagnosing, and monitoring diseases.^7,8^ Such metabolites are typically sampled from well-established matrices such as blood plasma and serum for trace-level analysis of up to thousands of metabolites using mass spectrometry (MS).^9^ Additional matrices of emerging interest for biomarker discovery and disease diagnosis applications include the rapid and noninvasive sampling of skin sebum and breath.^10−12^ Using MS, differences in the metabolite profiles in the blood plasma of pre-PD subjects were identified up to 15 years prior to a clinical diagnosis when compared to healthy controls who did not develop PD.^13^ These results suggest that PD may potentially be diagnosed using metabolite biomarkers significantly earlier than in current practice, particularly if analyzing such metabolites can result in high diagnostic accuracy and be validated on large-scale cohort studies.

To develop accurate prediction models for disease diagnosis using large metabolomics data sets, machine learning (ML) approaches are widely used.^14^ However, the use of whole metabolomics data sets to build prediction models is rare. Such data sets can contain highly correlated and “noisy” chemical features which may risk model overtraining and reduce diagnostic performance.^15^ As a result, models are typically based on a smaller subset of features which are determined by conventional statistical methods (e.g., based on *p*-values and fold-changes of individual features). For example, Gonzalez-Riano et al.^13^ used a linear support vector machine (SVM) model with 20 preselected biomarkers to diagnose pre-PD vs healthy controls from blood plasma samples. Similarly, Sinclair et al.^16^ used partial least-squares-discriminant analysis (PLS-DA) with 15 and 26 preselected biomarkers to diagnose drug naïve PD and medicated PD vs healthy control, respectively, from skin sebum samples. However, given that the abundances of metabolites are often correlated and can depend nonlinearly on the abundances of other metabolites,^17^ ML approaches such as SVM and PLS-DA may potentially “miss” some key features in metabolomics data sets.

Advanced ML approaches such as neural networks (NN) are particularly well-suited for processing large volumes of correlated data and building models for data sets that contain nonlinear effects.^18^ However, a fundamental issue in using methods such as NN for classifying complex mixtures based on metabolomics data is that the resulting predictive models are generally considered as uninterpretable “black boxes”, which cannot be readily used to reveal mechanistic information.^19,20^ Recently, a new approach entitled Shapley Additive exPlanations (SHAP) was developed to “interpret” ML models by retrospectively calculating the contribution of individual features to the accurate predictive performance of a model.^21^ However, SHAP has not been used in the analysis of metabolomics data sets given that methods for interpreting ML models have only recently been developed, and using all chemical features risks overtraining prediction models. Ideally, whole metabolomics data sets should be included in the ML model for SHAP to identify key metabolites that drive model prediction.

**Figure 1:**
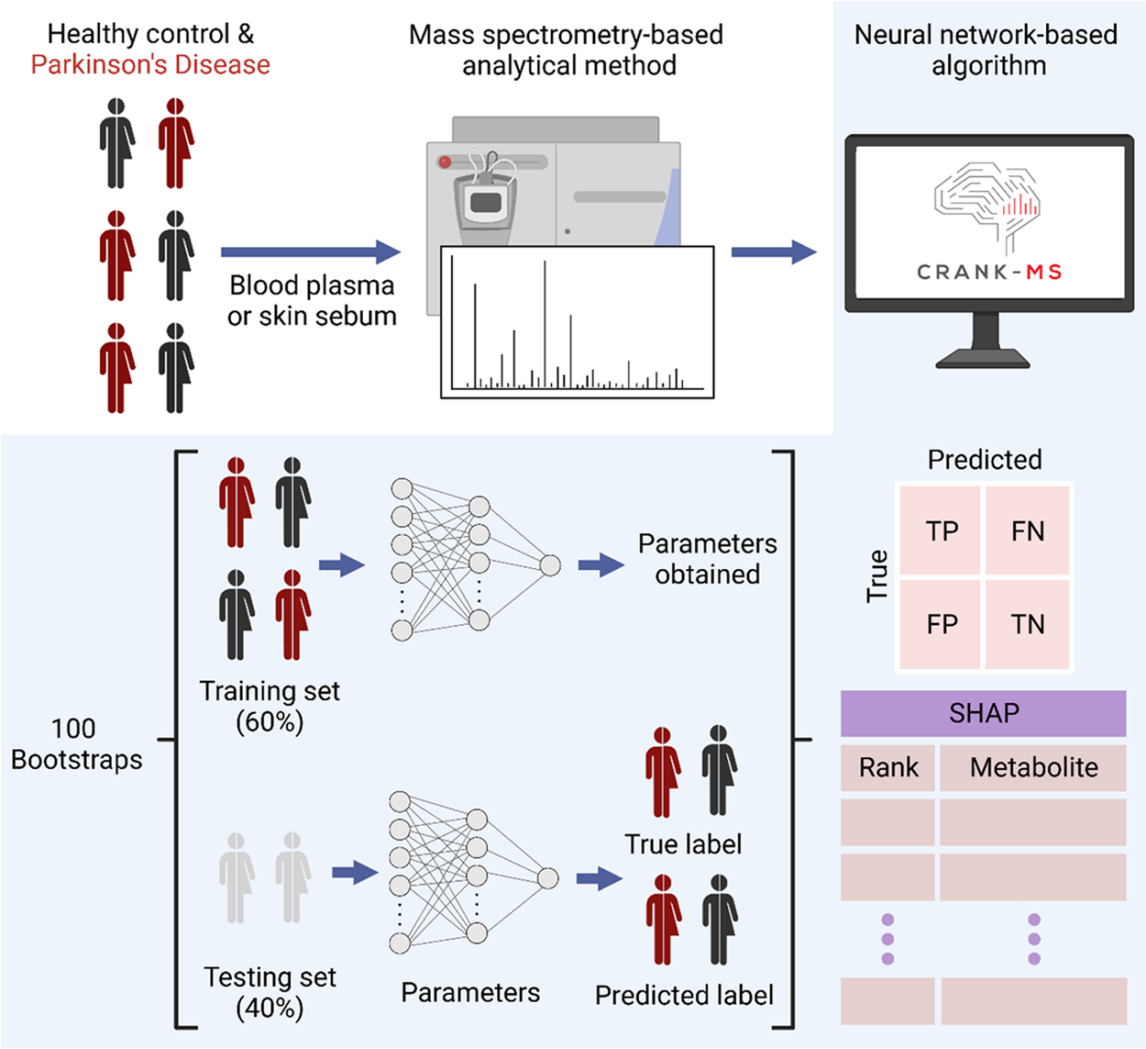
Neural network (NN) framework for predicting Parkinson’s disease using large mass spectrometry-based metabolomics data. Whole metabolomics data sets without feature selection can be analyzed directly by NN for the binary classification of Parkinson’s disease. Using a 100-iteration bootstrap model, 60% of the data was randomly distributed for training and 40% for testing. Diagnostic performance for each bootstrap was calculated based on the absolute values obtained for true positive (TP), false negative (FN), false positive (FP), and true negative (TN). SHapely Additive exPlanations (SHAP) analysis of NN was used to rank chemical features based on the extent of their contribution to a correct Parkinson’s disease prediction. Subjects can either have PD at the time of sample collection^16^ or later develop PD up to 15 years after sample collection^13^ depending on the study design.

Here, we report an interpretable neural network-based framework for analyzing data sets generated by untargeted mass spectrometry-based methods (Figure 1) entitled, “CRANK-MS” (Classification and Ranking Analysis using Neural network generates Knowledge from Mass Spectrometry). CRANK-MS has several built-in features including (i) integrated model parameters that allow the high dimensionality of metabolomics data sets to be analyzed without the need for preselecting chemical features; (ii) SHAP to retrospectively “mine” key chemical features that contribute the most to an accurate model prediction; and (iii) benchmark testing with five well-known ML methods to compare diagnostic performance and further verify significant chemical features. Using CRANK-MS, we report the highest diagnostic performance to date for binary classification of PD vs healthy control. Additionally, NN-driven predictions trained on a prognostic PD study were used to reveal new PD-specific chemical features which were not previously identified and can be considered indicative of pre-PD diagnosis. The program for implementing this approach is freely available online at https://github.com/CRANK-MS.

## Methods

### Data

Data sets from two cross-sectional PD metabolomics studies^13,16^ were used throughout. The Spanish European Prospective Study on Nutrition and Cancer (EPIC) study^13^ involved metabolomics data from blood plasma samples taken from subjects who later developed PD up to 15 years later, and those who did not develop PD (total number of participants, *n* = 78; Table 1). The blood plasma samples from the EPIC study were analyzed using four different instrumental methods (gas chromatography-MS, GC-MS; capillary electrophoresis-MS, CE-MS; and liquid chromatography-MS, LC-MS, in positive (+) and negative (−) ionization modes). The NHS study^16^ involved the LC-MS (+) analysis of skin sebum sampled from drug-naïve and medicated PD patients, and healthy controls (*n* = 274). The composite data set from the EPIC study was prepared using the data from all four methods (i.e., GC-MS, CE-MS, LC-MS (+), and LC-MS (−)). Six participants were excluded in the composite data set as data from one or more of the four methods were missing. The number of reported molecular features in the metabolomics data sets ranged from 60 to 6502 (Table 1).

**Table 1:** Summary of Demographic and Chemical Feature Information for Metabolomics Data

cohort	data set	*N* = Control	*N* = PD	biological sex	age	number of features	sample type
EPIC^13^^a^	GC-MS	39	36	Women (46%) and men (54%)	41–69 years old (at the time of sample collection)	60	Plasma
	CE-MS	39	39			329	Plasma
	LC-MS (+)	39	39			509	Plasma
	LC-MS (−)	37	36			532	Plasma
	Composite (GC-MS, CE, LC-MS (+), LC-MS (−)	37	35			1430	Plasma
NHS^16^^b^	LC-MS (+)	56	80^c^	Healthy control: women (54%) and men (46%)	Healthy control: 40–69 years old	6502	Sebum
	LC-MS (+)	56	138^d^	Drug-naïve PD: women (36%) and men (64%)	Drug naïve PD: 60–79 years old	6502	Sebum
				Medicated PD: women (38%) and men (62%)	Medicated PD: 62–79 years old		

a Blood samples from all participants were collected between 1993 and 1996 from 3 regions in Spain (Murcia, Navarra, and Gipuzkoa). Participants were randomly recruited from the general population and were considered healthy at the time of sample collection. Most participants were blood donors.^37^ Those who later developed PD were identified in a follow-up period where PD diagnosis was made between the time of recruitment up until 2011. Thus, this group of PD can be considered as pre-PD where PD diagnosis was made up to 15 years after the blood sample was collected. PD diagnosis was confirmed from various sources including primary health and hospital records. For many PD cases, a matched control was determined based on several factors including study recruitment center, age, and time of day during sample collection, and fasting period.^13^

b Skin sebum samples were collected from 25 recruitment sites across the United Kingdom and one recruitment site from The Netherlands.^16^ Any additional information regarding recruitment was not reported.

c Healthy control vs drug-naïve PD.

d Healthy control vs medicated PD.

### Machine Learning Algorithms

Using the metabolomics data as inputs, we leveraged six supervised learning frameworks to classify persons with PD from those who are healthy. Random forest (RF), extreme gradient boosting (XGB), linear discriminant analysis (LDA), logistic regression (LR), and support vector machine (SVM) were written using scikit-learn packages (v 1.0.2). The algorithms and SHAP analysis were implemented in Python (v. 3.8). NN with multilayer perceptron was written using PyTorch (v. 1.10.2). Additional Python libraries used to support data analysis and visualization include pandas (v. 1.4.2), numpy (v. 1.21.5), and matplotlib (v. 3.5.1). Full details of the open access code are available at https://github.com/CRANK-MS.

### Pseudocode

**Chart 1:**
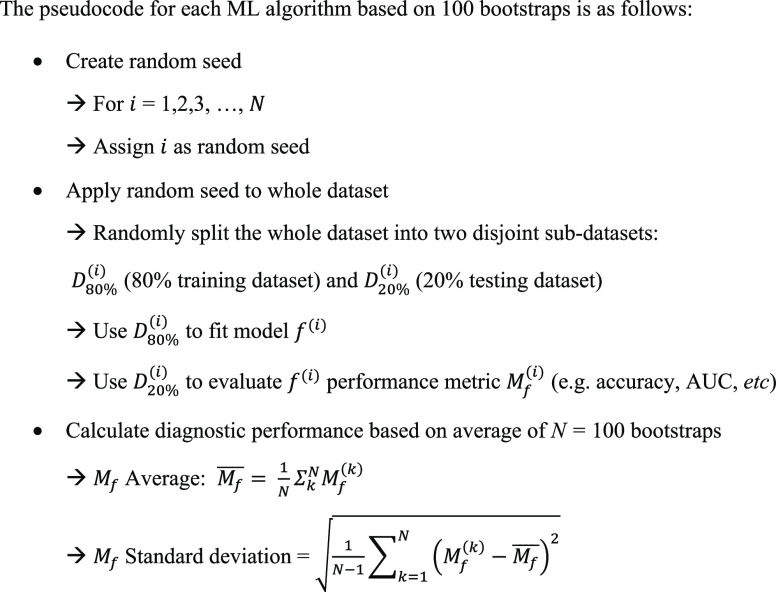


The pseudocode for each ML algorithm based on 100 bootstraps is presented in Chart 1.

### Hyperparameter Tuning

For each ML model, hyperparameters correspond to specific model parameters that an algorithm uses to train on a given data set. To determine the optimal hyperparameters, the composite data set from the EPIC study was used (see above). Hyperparameter tuning for each ML model was optimized using the *GridSearchCV* package in scikit-learn. For NN, XGB, RF, LR, and SVM, the number of possible permutations resulting from different parameters ranged from 120 to 158. For LDA, the number of permutations used was 12. For each permutation, a bootstrap model was used in which the data set was split randomly 100 times into 60% training data and 40% validation data (i.e., 100 “bootstraps”). The optimized hyperparameters were determined based on the combination of hyperparameters that resulted in the highest Matthews correlation coefficient (MCC) after 100 iterations per permutation. These hyperparameters were then applied to all data sets in the study (Table S1).

### Performance Metrics

To calculate diagnostic performance, the 100 randomly selected testing data sets from the bootstrapping process were used to calculate the overall diagnostic performance. The use of a bootstrap model with more replicates can lower absolute error compared to other sampling methods such as cross-validation and is considered useful for relatively small sample sizes.^22,23^ The diagnostic performance for each ML model was calculated based on the mean of the 100 bootstrap measurements, and error was calculated as one standard deviation of the mean. For each ML model, accuracy, precision, sensitivity/recall, specificity, *F*1 score, and MCC score were calculated. Receiver operating characteristic (ROC) and precision-recall (PR) curves were generated to calculate area-under-curve (AUC). Briefly, AUC (ROC) is a plot of the true positive rate (i.e., how many PD patients were correctly predicted) vs the true negative rate (i.e., how many healthy controls were correctly predicted). In contrast, AUC (PR) plots the precision rate (i.e., how many PD predictions were correct) vs recall or sensitivity rate (i.e., how many PD patients were correctly predicted).

### Annotation of Chemical Features

For each chemical feature in the metabolomics data sets, a SHAP score was calculated based on the absolute average from the 100 bootstraps. The greater the SHAP scores, the greater the contribution to the overall prediction of PD across all 100 bootstraps and all participants. Metabolites were annotated based on the highest mass accuracies that were obtained by comparing the measured monoisotopic neutral masses (accounting for protonation, sodiation, and potential loss of a water molecule) to those from the Human Metabolome Database (https://hmdb.ca/) using a threshold of ±20 ppm. One top scoring chemical feature (*m*/*z* 942.9824) had a relatively large negative mass defect which is indicative of an exogenous, synthetic compound. Thus, this ion was annotated using the PubChem (http://www.cheminfo.org/) database.

## Results and Discussion

### Highest Diagnostic Accuracy to Date for PD Using Metabolomics: NN Outperforms Other ML Algorithms

**Figure 2:**
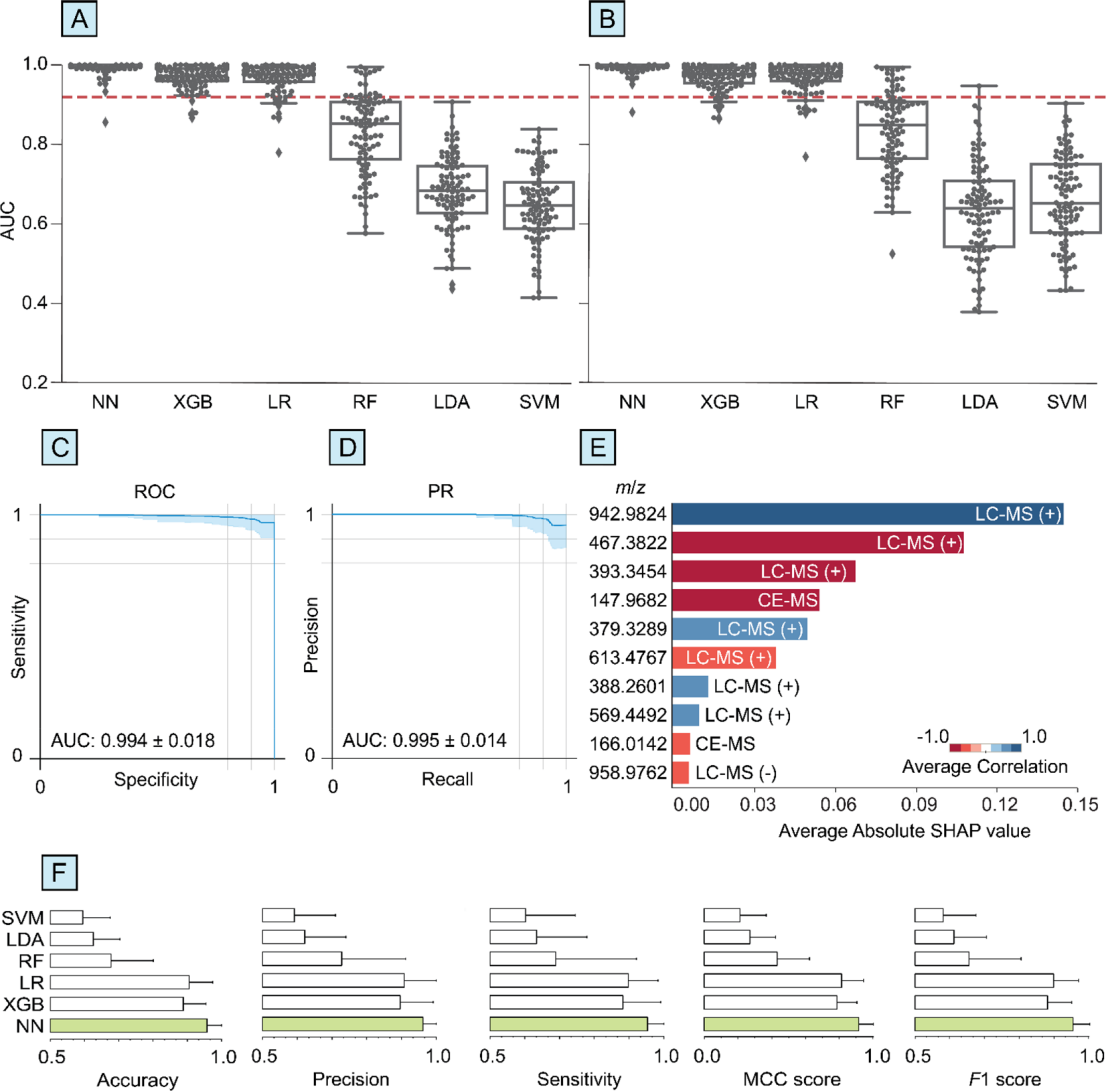
The use of neural networks (NN) can outperform other machine learning algorithms for the early diagnosis of Parkinson’s disease using blood plasma metabolomics data. A composite data set from the EPIC study was used involving metabolomics data from liquid chromatography–mass spectrometry (LC-MS) in positive and negative ionization modes, capillary electrophoresis-mass spectrometry (CE-MS), and gas chromatography-MS (GC-MS) without any feature selection.^13^ Box-swarm plots of area under curves (AUC) of (A) receiver-operating curve (ROC) and (B) precision-recall (PR) for NN, extreme gradient boosting (XGB), logistic regression (LR), random forest (RF), linear discriminant analysis (LDA), and support vector machine (SVM) classifiers. The red line corresponds to the AUC previously reported^13^ based on a feature-selected SVM classifier. Overall (C) ROC and (D) PR plots are shown for NN. (E) Shapely additive explanations (SHAP) values for NN for the top ions (*m*/*z*) that had the highest contribution to a correct PD prediction and the corresponding analytical method. The average correlation corresponds to whether the feature is up- (blue) or down- (red) regulated. For each algorithm, (F) accuracy, precision, sensitivity, MCC score, and *F*1 score are shown (green bars correspond to the highest performance obtained by NN).

The overall diagnostic performance for all six ML algorithms was assessed using a composite data set featuring metabolites from blood plasma that were detected using four analytical methods as reported in the EPIC study.^13^ The diagnostic performance of NN was higher than the other frameworks across all metrics investigated (Figure 2, Table S2). Specifically, the binary classification of PD vs healthy using NN resulted in AUCs of 0.994 ± 0.018 and 0.995 ± 0.014 for ROC and PR, respectively. Extreme gradient boosting and logistic regression performed similarly with AUC (ROC) and AUC (PR) of 0.970 ± 0.028 and 0.968 ± 0.031 for extreme gradient boosting, and 0.968 ± 0.037 and 0.969 ± 0.037 for logistic regression, respectively. In contrast, the performance of the RF, SVM, and LDA classifiers were relatively low with AUC (ROC) and AUC (PR) values of 0.829 ± 0.099 and 0.836 ± 0.099 for the RF classifier, 0.647 ± 0.093 and 0.661 ± 0.111 for the SVM classifier, and 0.681 ± 0.091 and 0.634 ± 0.119 for the LDA classifier, respectively. Additional metrics, i.e., accuracy, sensitivity, specificity, precision, and *F*1 score can be found in Table S2. For all six algorithms, randomly permuting the PD and healthy data labels resulted in average accuracies (Figure S1) that are statistically the same as 0.5 (50%), consistent with a “random guess” for binary classification as expected for this control test. Recently, Chicco et al.^24^ has shown that the MCC score is a more informative and reliable metric for evaluating binary classification accuracy as it considers all four values in the confusion matrix (i.e., true positive, false positive, true negative, and false negative). Thus, MCC can be considered as a less biased metric toward data sets with imbalanced cohorts. Based on the MCC score, NN performed significantly higher with 0.918 ± 0.086 compared to 0.815 ± 0.132, 0.787 ± 0.119, 0.433 ± 0.192, 0.272 ± 0.152, and 0.213 ± 0.155 for the LR, XGB, RF, LDA, and SVM classifiers, respectively.

**Figure 3:**
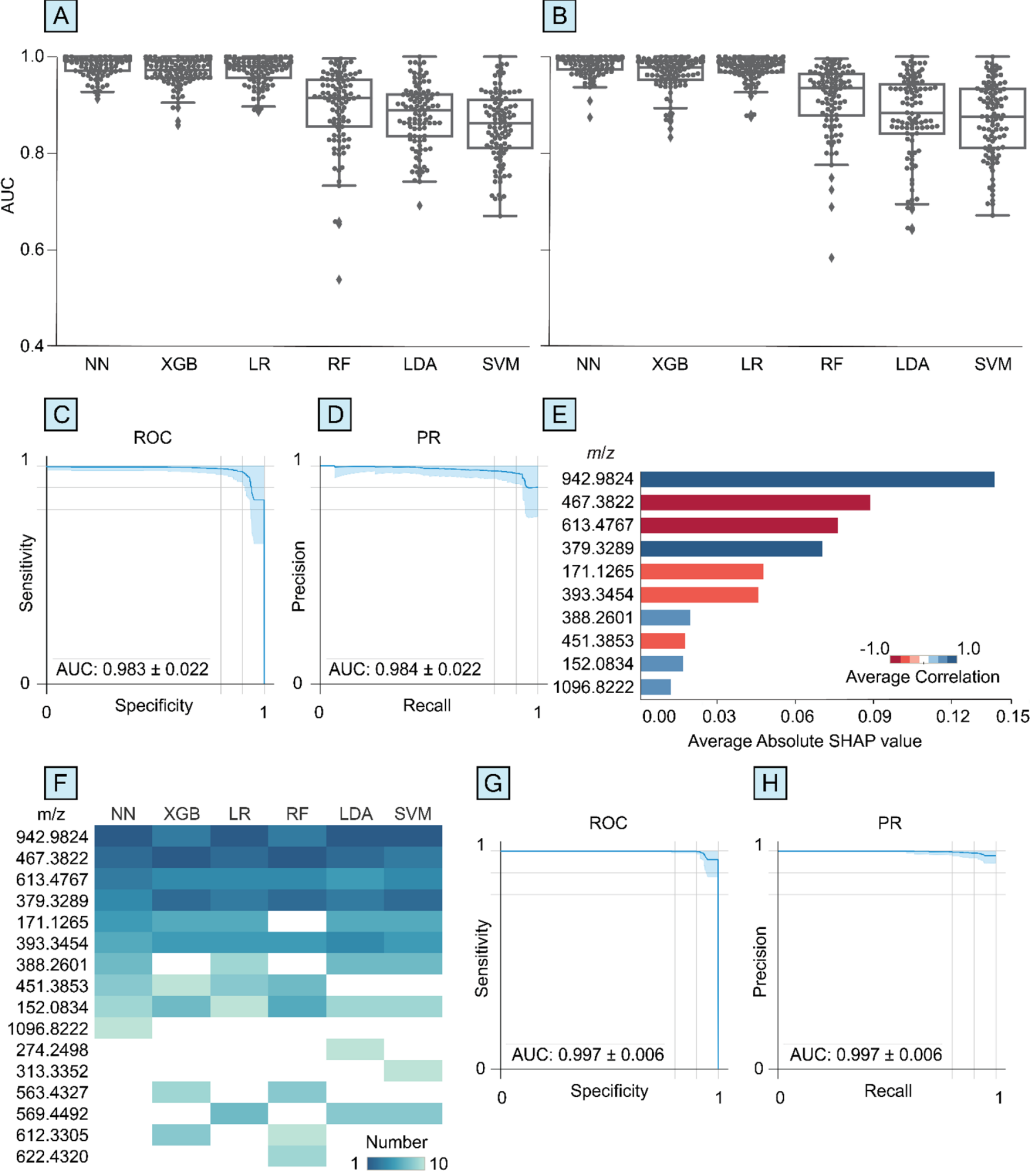
The use of neural networks (NN) resulted in the highest performance for diagnosing Parkinson’s disease from blood plasma metabolomics data (EPIC study^13^) obtained by liquid chromatography–mass spectrometry in positive mode (LC-MS (+)) without any preselection of chemical features. Box-swarm plots of area under curves (AUC) for (A) receiver-operating curve (ROC) and (B) precision-recall (PR) for NN, extreme gradient boosting (XGB), logistic regression (LR), random forest (RF), linear discriminant analysis (LDA), and support vector machine (SVM) classifiers. Overall (C) ROC and (D) PR plots are shown for NN. (E) Shapely additive explanations (SHAP) values for NN shown for the top ten ions (*m*/*z*) using LC-MS (+) that had the highest contribution to a correct PD prediction. The average correlation corresponds to whether the feature is up- (blue) or down- (red) regulated. (F) Comparative SHAP values and relative rankings for the top ten metabolites for all six ML algorithms. (G) ROC and (H) PR plots are shown for a feature-selected NN-model using the top ten metabolites identified from SHAP.

SHAP analysis was used to identify the metabolites and the corresponding mass spectrometry-based method that contributed the most to the prediction of PD using the composite metabolomics data set for blood plasma. Five of the top six metabolites were detected using LC-MS (+) (i.e., *m*/*z* 942.9824, 467.3822, 393.3454, 379.3289, and 613.4767) (Figure 2). To further validate the contribution of these chemical features in predicting PD, all six ML algorithms were applied to the LC-MS (+) data set without including the LC-MS (−), GC-MS, and CE-MS data sets. Similar to that for the composite data set, binary classification of PD using NN and the LC-MS (+) data was highest across all performance metrics (Figures 3 and S2, Table S2). For example, the AUC (ROC), AUC (PR), and MCC score for NN was 0.983 ± 0.022, 0.984 ± 0.022, and 0.894 ± 0.081, respectively. Both XGB and LR classifiers performed about the same or slightly lower with AUC (ROC), AUC (PR), and MCC values of 0.968 ± 0.029, 0.966 ± 0.036, 0.805 ± 0.117, and 0.972 ± 0.029, 0.976 ± 0.026, 0.869 ± 0.078, respectively. RF, SVM, and LDA classifiers were substantially lower with AUC (ROC), AUC (PR), and MCC scores of 0.894 ± 0.079, 0.911 ± 0.071, 0.589 ± 0.177 for the RF classifier; 0.856 ± 0.073, 0.869 ± 0.078, 0.582 ± 0.167 for the SVM classifier; and 0.878 ± 0.066, 0.877 ± 0.086, and 0.626 ± 0.150 for the LDA classifier. Additional metrics are given in Table S2. The standard deviations for all performance metrics were pooled to calculate the average relative standard deviation (RSD). The RSD was lowest for NN (5.17%) followed by LR (5.19%), XGB (7.70%), LDA (12.8%), SVM (14.3%), and RF (17.2%). Based on a SHAP analysis, five of the six top-scoring chemical features in the LC-MS (+) data set were also in the top six highest scoring features for the composite data set (see above, Figure 3). These results indicate that the diagnostic performance for the LC-MS (+) data set outperformed the three other mass spectrometry-based methods across all six ML methods. The use of NN resulted in higher diagnostic accuracy than the other five ML methods for the LC-MS (+) data set, which has significantly fewer chemical features (509) than in the composite data set (1430).

Overall, NN resulted in the highest diagnostic performance and the lowest RSD in predicting PD from blood plasma using either the composite or LC-MS (+) data sets compared to the other five ML algorithms. Disease classification using NN involving 100 bootstraps and 1430 total metabolites required <1 min on a consumer laptop computer (Surface Laptop 3, Microsoft) with a 1.2 GHz processor (Intel i5-core). The diagnostic performance of NN was at least 10% higher than previously reported by Gonzalez-Riano et al. when a SVM model was used.^13^ In addition, the diagnostic performance obtained using NN is the highest reported to date for any PD diagnosis regardless of the sample matrix including blood plasma,^25,26^ blood serum,^27^ and skin sebum.^10,11,16,28,29^

### Diagnostic Performance Is Uncompromised by Including the Whole Metabolomics Dataset

The NN model included all metabolites or features from the data set as inputs unlike training models with preselected chemical features. For example, in Gonzalez-Riano et al.,^13^ biomarkers for PD were first screened for significance, and a small subset of these biomarkers (up to 20) was used in the final diagnostic model. In this previous study, the highest ROC (AUC) value obtained was 0.919 for the composite data set using a 20-feature linear SVM model.^13^ In the current study, a similar model was applied to the composite data set without feature selection which resulted in an AUC (ROC) of 0.647 ± 0.093. In contrast, using NN on all features in the composite data set resulted in an AUC (ROC) of 0.994 ± 0.018. These results are consistent with some well-known ML models having relatively low predictive performance when incorporating large data sets that contain many “noisy” features.

A feature-selected NN model was developed using the ten chemical features that had the highest SHAP scores in the LC-MS (+) data set. The AUC ROC and PR values for the feature-selected NN-model were comparable or slightly higher (0.997 ± 0.006 and 0.997 ± 0.006) than that obtained using all chemical features in the data set (0.983 ± 0.022 and 0.984 ± 0.022). Given that the diagnostic performance of both models was comparable, these data indicate that NN can be highly tolerant of many chemical features (>1500) that do not contribute substantially to accurate disease prediction; i.e., diagnostic performance is essentially uncompromised by including the whole metabolomics data set without feature selection. In addition, the relatively high diagnostic accuracy supports the use of SHAP to accurately identify chemical features that contribute significantly to disease classification.

### Revealing New Metabolite Biomarkers for PD by Retrospective Analysis

The metabolites that contributed the most significantly to the accurate prediction of PD are more likely to be more basic and readily ionized by cation adduction, rather than acidic, given that higher diagnostic performance for PD was obtained using LC-MS (+) than LC-MS (−) and that similar numbers of metabolites (∼510 to 530) were measured using each method, consistent with previous reports.^25,27^ SHAP analysis on LC-MS (+) data for blood plasma revealed that five of the top six highest scoring metabolites were consistent across all six ML algorithms (Figure 3). The detected metabolites were different compared to those determined using a previous study focused on a linear SVM model^13^ (Figure S3), which can be attributed to the difference between using kernel-based methods and neural networks.

The five metabolites that contributed the most to an accurate prognostic PD prediction could serve as potential indicators for disease status and were annotated (Table 2). The five annotated ions corresponded to a polyfluoroalkyl substance (PFAS), triterpenoids, cholestane steroids, diacylglycerol, and vitamin D steroids of either endogenous or exogenous origins, which have been linked to PD in the literature previously (Table 2). For example, the ion with an *m*/*z* value of 942.9824 had the highest SHAP value and likely corresponds to the sodiated PFAS [3-(2,2,3,3,4,4,4-heptafluorobutanoyloxy)-2,2-bis(2,2,3,3,4,4,4-heptafluorobutanoyloxymethyl)propyl] 2,2,3,3,4,4,4-heptafluorobutanoate (DTXSID70325550). Ions corresponding to this PFAS and its oxidation ([3-(2,2,3,3,4,4,4-heptafluorobutanoyloxy)-2,2-bis(2,2,3,3,4,4,4-heptafluorobutanoyloxymethyl)-1-hydroxypropyl] 2,2,3,3,4,4,4-heptafluorobutanoate) and hydrolysis (i.e., 2,2-bis(hydroxymethyl)propane-1,3-diol) products were all higher in PD participants than healthy controls. The presence of PFAS compounds are ubiquitous in the environment and human blood given their propensity to bioaccumulate, chemical longevity, and widespread use in industrial and consumer products such as plastics, nonstick cookware, and food packaging.^30,31^ For example, in the U.S. population, PFAS was detected in the blood serum of over 98% of Americans that were sampled during 2003–2004 (*n* = 2,094).^32^ DTXSID70325550 is a PFAS compound of interest that is currently listed under the U.S. Environmental Protection Agency CompTox Chemicals Database^33^ and appears preorganized for the noncovalent, multidentate binding of Na^+^, K^+^, Ca^2+^, Cu^2+^, and Zn^2+^. Thus, such a compound could potentially disrupt neuronal activity by affecting intracellular ion homeostasis.^34,35^ A potential mechanism proposed for PFAS-induced neurotoxicity involves the increase of intracellular Ca^2+^ which is implicated in impacting neuronal cell processing, signaling, and function.^30,35^ Although further *in vitro* and *in vivo* studies are needed to investigate the effects of DTXSID70325550 on neuronal cell function, these data suggest that elevated levels of specific PFAS compounds in blood plasma may be an early indicator of PD. Overall, these results further support that SHAP analysis can be useful in identifying potential biomarkers for PD (Table 2) that were not initially found using statistical approaches.^13^

**Table 2:** Summary of Five Annotated Metabolites That Contributed Most to a Parkinson’s Disease Prediction

*m*/*z*	compound class	annotation	chemical formula	ion	up-/down-regulated	*p*-value	link to PD
942.9824	Polyfluorinated alkyl substance	[3-(2,2,3,3,4,4,4-heptafluorobutanoyloxy)-2,2-bis(2,2,3,3,4,4,4-heptafluorobutanoyloxymethyl)propyl] 2,2,3,3,4,4,4-heptafluorobutanoate	C_21_H_8_F_28_O_8_	[M + Na]^+^	Up	5.4 × 10^–11^	A proposed mechanism for PFAS-induced neurotoxicity involves the increase of intracellular Ca^2+^ which is implicated in impacting neuronal cell processing, signaling, and function.^30,35^ Noncovalent binding of metal ions by this PFAS could disrupt neuronal activity by affecting intracellular ion homeostasis.^34,35^
467.3822	Triterpenoid	Dammarenediol II^a^	C_30_H_52_O_2_	[M + Na]^+^	Down	3.1 × 10^–09^	Triterpenoids have been linked to the activation of the nuclear factor-E2-related factor-2 (Nrf2)/antioxidant response element (ARE) signaling pathway which regulates oxidative stress.^38−40^ Oxidative stress is a leading factor in the pathogenesis of PD which includes dopaminergic cell death, mitochondrial dysfunction, and inflammation.^41^ Triterpenoids can be consumed through food sources including apple, olive, tomato, and soybean.^39,42,43^
613.4767	Diacylglycerol	1,2-diacylglycerol (34:3) isomers^b^	C_37_H_66_O_5_	[M + Na]^+^	Down	3.2 × 10^–06^	Diacylglycerols are naturally found in vegetable oils such as olive oil,^44^ where consumption of unsaturated lipids is an important component in a Mediterranean diet.^36^ A recent study by Barbalace et al.^45^ reported that extra virgin olive oil extract can significantly increase the brain-derived neurotrophic factor, which is a key signaling pathway for neuronal survival, regulation, and regeneration.^46,47^
379.3289	Steroid	Vitamin D2^c^	C_28_H_44_O	[M + H – H_2_O]^+^	Up	4.5× 10^–07^	The presence of Vitamin D has previously been implicated as biomarkers in PD.^13,48^
393.3454	Cholestane steroid	Cholest-5-ene	C_27_H_46_	[M + Na]^+^	Down	1.1 × 10^–06^	Cholestane derivatives have been shown to have neuroprotectant properties.^49^ For example, using animal models, Hu et al.^50^ reported that an endogenous cholestane derivative could directly block NMDA receptors where overactivation of these receptors is typically observed in PD.^51^

a See Table S3 for other potential triterpenoid isomers listed in the HMDB that have not been detected in blood, unlike Dammarenediol II.

b 1,3-Diacylglycerol (34:3) isomers were also listed in the HMDB.

c Vitamin D2 agrees with the annotation by Gonzalez-Riano et al.^13^ based on the EPIC cohort. See Table S3 for other potential steroid isomers listed in the HMDB.

### NN Resulted in Higher Performance for Diagnosing PD than Other ML Methods Using a Larger Metabolomics Dataset from Sebum Samples

**Figure 4:**
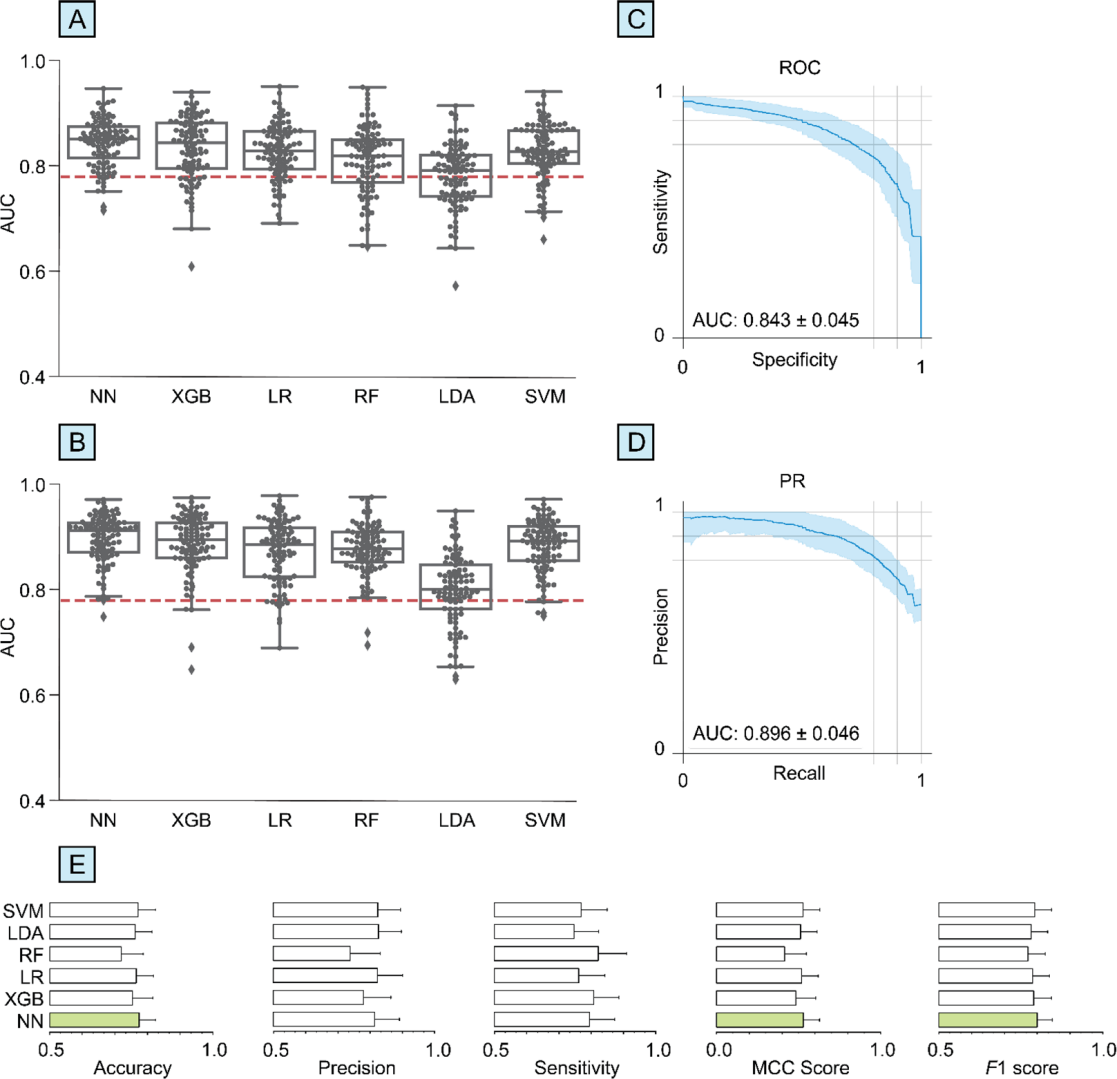
Neural network (NN) can result in higher overall diagnostic performance for drug-naïve PD vs healthy control from metabolomics data (NHS PD study^16^) of skin sebum samples without any selection of >6500 chemical features. Box-swarm plots of area under curves (AUC) of (A) receiver-operating curve (ROC) and (B) precision-recall (PR) for NN, extreme gradient boosting (XGB), logistic regression (LR), random forest (RF), linear discriminant analysis (LDA), and support vector machine (SVM) classifiers. The red line corresponds to the AUC previously reported^16^ based on a feature-selected partial least-squares-discriminant analysis classifier. ROC and PR plots are shown for NN in (C) and (D), respectively. For each algorithm, (E) accuracy, precision, sensitivity, MCC score, and *F*1 score are shown (green bars correspond to the highest performance obtained by NN).

The performance of CRANK-MS was assessed on a larger sample size with more chemical features. The six ML algorithms were used to analyze the data from the NHS study^16^ to predict PD patients that were drug-naïve or medicated from healthy controls using LC-MS (+) metabolomics data from skin swab samples of sebum. On average across all performance metrics, the performance of NN was higher than the five other algorithms (Figure 4, Table S2). Binary classification of drug-naïve PD vs healthy control using NN resulted in AUC (ROC), AUC (PR), and MCC scores of 0.843 ± 0.045, 0.896 ± 0.046, and 0.530 ± 0.098 respectively. Additional performance metrics are shown in Table S2. In addition, the average RSD was lowest for NN at 9.51% followed by SVM (9.63%), LR and LDA (10.0%), XGB (11.7%), and RF classifiers (14.0%). The accuracy in predicting medicated PD vs healthy control across all six algorithms was about the same or slightly higher compared to drug-naïve PD vs healthy control (Table S2), consistent with the medicated PD cohort being more accurately diagnosed based on a clinical assessment of their symptoms and/or medication influencing skin sebum metabolites.

For the drug-naïve PD vs healthy control data set, the diagnostic performance obtained using NN on skin sebum was more than 8% higher than previously reported by Sinclair et al.^16^ which used a multivariate principle least-squares-discriminant analysis model based on 15 preselected features. Specifically, the AUC (ROC) using multivariate principle least-squares-discriminant analysis was 0.779 compared to 0.843 ± 0.045 using NN.^16^ The NN approach required no preselection of features and took 10 min to obtain the key diagnostic performance metrics (i.e., accuracy, sensitivity, specificity, precision, *F*1 score, MCC score, AUC ROC, AUC PR) using all 6,502 chemical features in the data set. The relatively lower diagnostic performance obtained for the NHS study compared to the EPIC study may be attributable to different study designs (Table 1) in addition to differences in the use of blood plasma vs skin sebum. For example, in the EPIC study, PD participants were matched to healthy subjects based on several factors including the same recruitment center, age, and time of day for sample collection, and fasting period, unlike in the NHS study.

## Conclusions

In this study, a NN framework with interpretable feature analysis, entitled CRANK-MS, is reported that can be used to establish accurate disease prediction models using whole MS data sets without preselecting features. The NN is highly tolerant of “noisy” metabolomics data that can contain thousands of metabolites which may not contribute significantly to model prediction. Using CRANK-MS, we report the highest diagnostic performance to date for predicting PD using blood plasma metabolomics data (0.997 AUC for both ROC and PR) when benchmarked with well-known ML algorithms such as PLS-DA and SVM. Diagnostic accuracy in predicting PD using skin sebum metabolomics data was also enhanced using NN compared to alternative, widely used ML approaches. PD-specific biomarkers including triterpenoids, diacylglycerols, and a polyfluoroalkyl substance that contributed significantly to ML model predictions were identified from blood samples that were collected up to 15 years prior to when subjects were clinically diagnosed with PD. These data indicate that these metabolites are potential early indicators for PD that predate clinical PD diagnosis and are consistent with specific food diets (such as the Mediterranean diet^36^) for PD prevention and that exposure to some exogenous chemicals (such as PFASs that can disrupt neuronal activity via changes to intracellular ion homeostasis^34,35^) may contribute to the development of PD.

Given the improved diagnostic performance of CRANK-MS, it is anticipated that this NN-based framework can be a powerful tool to build accurate prediction models for other diseases using metabolomics data. Interpretable ML methods can also be used to retrospectively “mine” metabolomics data sets to identify early “lead” compounds within the biomarker discovery pipeline. Biomarkers identified using this approach can be further validated using high-resolution tandem mass spectrometry and NMR for complete structure elucidation and targeted quantification using clinical MS-based methods, in addition to using *in vitro* cell-based assays and *in vivo* disease models. For example, future studies on targeted quantification of pre-PD metabolites could be used to track and monitor disease progression by integrating data on clinical deterioration and other factors such as lifestyle, medication, and diet. In addition, given that there are over 800 publicly available metabolomics studies in The Metabolomics Workbench data repository (https://www.metabolomicsworkbench.org/), prediction models could be used for the binary classification of diseases such as diabetes, fatty liver disease, heart disease, chronic obstructive pulmonary disease, and COVID-19. Using advanced ML methods, retrospectively “mining” such databases for biomarkers that contribute significantly to the prediction of these diseases could reveal novel mechanistic information that may not necessarily be apparent using traditional linear approaches. In addition, CRANK-MS could be adapted to support clinical workflows and improve confidence in diagnosis including for diseases in which stratification is important by performing multinomial classification (>2 classes). For example, CRANK-MS can be used with metabolomics data in conjunction with alternative clinical information such as medical history and neurological examination scores, as well as neuroimaging, to further differentiate clinical PD from other types of Parkinsonian-like diseases. Ultimately, the use of CRANK-MS should enhance the accuracy of disease prediction models based on metabolomics and many other types of ‘omics experiments and facilitate biomarker discovery.

## Supporting Information Available

Permutation tests for each ML method using the composite data set; AUC ROC and PR box-swarm plots for different ML and MS-based methods; volcano plot of top scoring metabolites; hyperparameter values for each ML method; table summarizing all performance metrics and data sets; and list of potential isomers (PDF)

## References

[ref1] Feigin V. L.; et al. Global, regional, and national burden of neurological disorders during 1990–2015: a systematic analysis for the Global Burden of Disease Study 2015. Lancet Neurology 2017, 16 (11), 877–897. 10.1016/S1474-4422(17)30299-5. 28931491 PMC5641502

[ref2] Armstrong M. J.; et al. Diagnosis and Treatment of Parkinson Disease: A Review. JAMA 2020, 323 (6), 548–560. 10.1001/jama.2019.22360. 32044947

[ref3] Chaudhuri K. R.; et al. Non-motor symptoms of Parkinson’s disease: dopaminergic pathophysiology and treatment. Lancet Neurology 2009, 8 (5), 464–474. 10.1016/S1474-4422(09)70068-7. 19375664

[ref4] Poewe W.; Seppi K.; Tanner C. M.; Halliday G. M.; Brundin P.; Volkmann J.; Schrag A.-E.; Lang A. E. Parkinson disease. Nature Reviews Disease Primers 2017, 3 (1), 17013 10.1038/nrdp.2017.13. 28332488

[ref5] Hawkes C. H.; et al. A timeline for Parkinson’s disease. Parkinsonism & Related Disorders 2010, 16 (2), 79–84. 10.1016/j.parkreldis.2009.08.007. 19846332

[ref6] Rizzo G.; et al. Accuracy of clinical diagnosis of Parkinson disease. Neurology 2016, 86 (6), 566 10.1212/WNL.0000000000002350. 26764028

[ref7] Gowda G. A. N.; et al. Metabolomics-based methods for early disease diagnostics. Expert Review of Molecular Diagnostics 2008, 8 (5), 617–633. 10.1586/14737159.8.5.617. 18785810 PMC3890417

[ref8] Griffiths W. J.; et al. Targeted Metabolomics for Biomarker Discovery. Angew. Chem., Int. Ed. 2010, 49 (32), 5426–5445. 10.1002/anie.200905579. 20629054

[ref9] Dunn W. B.; et al. Systems level studies of mammalian metabolomes: the roles of mass spectrometry and nuclear magnetic resonance spectroscopy. Chem. Soc. Rev. 2011, 40 (1), 387–426. 10.1039/B906712B. 20717559

[ref10] Sinclair E.; et al. Validating Differential Volatilome Profiles in Parkinson’s Disease. ACS Central Science 2021, 7 (2), 300–306. 10.1021/acscentsci.0c01028. 33655068 PMC7908024

[ref11] Trivedi D. K.; et al. Discovery of Volatile Biomarkers of Parkinson’s Disease from Sebum. ACS Central Science 2019, 5 (4), 599–606. 10.1021/acscentsci.8b00879. 31041379 PMC6487537

[ref12] Hanna G. B.; et al. Accuracy and Methodologic Challenges of Volatile Organic Compound–Based Exhaled Breath Tests for Cancer Diagnosis: A Systematic Review and Meta-analysis. JAMA Oncology 2019, 5 (1), e182815–e182815. 10.1001/jamaoncol.2018.2815. 30128487 PMC6439770

[ref13] Gonzalez-Riano C.; Saiz J.; Barbas C.; Bergareche A.; Huerta J. M.; Ardanaz E.; Konjevod M.; Mondragon E.; Erro M. E.; Chirlaque M. D.; Abilleira E.; Goni-Irigoyen F.; Amiano P. Prognostic biomarkers of Parkinson’s disease in the Spanish EPIC cohort: a multiplatform metabolomics approach. npj Parkinson’s Disease 2021, 7 (1), 73 10.1038/s41531-021-00216-4. PMC8368017 34400650

[ref14] Liebal U. W.; Phan A. N. T.; Sudhakar M.; Raman K.; Blank L. M. Machine Learning Applications for Mass Spectrometry-Based Metabolomics. Metabolites 2020, 10 (6), 243 10.3390/metabo10060243. 32545768 PMC7345470

[ref15] Worley B. Multivariate Analysis in Metabolomics. Current Metabolomics 2013, 1 (1), 92–107. 10.2174/2213235X11301010092. 26078916 PMC4465187

[ref16] Sinclair E.; Trivedi D. K.; Sarkar D.; Walton-Doyle C.; Milne J.; Kunath T.; Rijs A. M.; de Bie R. M. A.; Goodacre R.; Silverdale M.; Barran P. Metabolomics of sebum reveals lipid dysregulation in Parkinson’s disease. Nat. Commun. 2021, 12 (1), 1592 10.1038/s41467-021-21669-4. 33707447 PMC7952564

[ref17] Camacho D.; et al. The origin of correlations in metabolomics data. Metabolomics 2005, 1 (1), 53–63. 10.1007/s11306-005-1107-3.

[ref18] Gardner M. W.; et al. Artificial neural networks (the multilayer perceptron)—a review of applications in the atmospheric sciences. Atmos. Environ. 1998, 32 (14), 2627–2636. 10.1016/S1352-2310(97)00447-0.

[ref19] Rudin C. Stop explaining black box machine learning models for high stakes decisions and use interpretable models instead. Nature Machine Intelligence 2019, 1 (5), 206–215. 10.1038/s42256-019-0048-x. PMC9122117 35603010

[ref20] Watson D. S.; et al. Clinical applications of machine learning algorithms: beyond the black box. BMJ. 2019, 364, l886 10.1136/bmj.l886. 30862612

[ref21] Qiu S.; et al. Multimodal deep learning for Alzheimer’s disease dementia assessment. Nat. Commun. 2022, 13 (1), 3404 10.1038/s41467-022-31037-5. 35725739 PMC9209452

[ref22] Smith G. C. S.; et al. Correcting for Optimistic Prediction in Small Data Sets. American Journal of Epidemiology 2014, 180 (3), 318–324. 10.1093/aje/kwu140. 24966219 PMC4108045

[ref23] Molinaro A. M.; et al. Prediction error estimation: a comparison of resampling methods. Bioinformatics 2005, 21 (15), 3301–3307. 10.1093/bioinformatics/bti499. 15905277

[ref24] Chicco D.; Jurman G. The advantages of the Matthews correlation coefficient (MCC) over F1 score and accuracy in binary classification evaluation. BMC Genomics 2020, 21 (1), 6 10.1186/s12864-019-6413-7. 31898477 PMC6941312

[ref25] Zhao H.; Wang C.; Zhao N.; Li W.; Yang Z.; Liu X.; Le W.; Zhang X. Potential biomarkers of Parkinson’s disease revealed by plasma metabolic profiling. Journal of Chromatography B 2018, 1081–1082, 101–108. 10.1016/j.jchromb.2018.01.025. 29518718

[ref26] Shao Y.; Li T.; Liu Z.; Wang X.; Xu X.; Li S.; Xu G.; Le W. Comprehensive metabolic profiling of Parkinson’s disease by liquid chromatography-mass spectrometry. Molecular Neurodegeneration 2021, 16 (1), 4 10.1186/s13024-021-00425-8. 33485385 PMC7825156

[ref27] Pereira P. A. B.; Trivedi D. K.; Silverman J.; Duru I. C.; Paulin L.; Auvinen P.; Scheperjans F. Multiomics implicate gut microbiota in altered lipid and energy metabolism in Parkinson’s disease. npj Parkinson’s Disease 2022, 8 (1), 39 10.1038/s41531-022-00300-3. PMC9001728 35411052

[ref28] Uehara Y.; Ueno S.-I.; Amano-Takeshige H.; Suzuki S.; Imamichi Y.; Fujimaki M.; Ota N.; Murase T.; Inoue T.; Saiki S.; Hattori N. Non-invasive diagnostic tool for Parkinson’s disease by sebum RNA profile with machine learning. Sci. Rep. 2021, 11 (1), 18550 10.1038/s41598-021-98423-9. 34545158 PMC8452747

[ref29] Fu W.; et al. Artificial Intelligent Olfactory System for the Diagnosis of Parkinson’s Disease. ACS Omega 2022, 7 (5), 4001–4010. 10.1021/acsomega.1c05060. 35155895 PMC8829950

[ref30] Cao Y.; et al. Absorption, distribution, and toxicity of per- and polyfluoroalkyl substances (PFAS) in the brain: a review. Environmental Science: Processes & Impacts 2021, 23 (11), 1623–1640. 10.1039/D1EM00228G. 34533150

[ref31] Foguth R.; et al. Per- and Polyfluoroalkyl Substances (PFAS) Neurotoxicity in Sentinel and Non-Traditional Laboratory Model Systems: Potential Utility in Predicting Adverse Outcomes in Human Health. Toxics 2020, 8 (2), 42 10.3390/toxics8020042. 32549216 PMC7355795

[ref32] Calafat A. M.; Wong L.-Y.; Kuklenyik Z.; Reidy J. A.; Needham L. L. Polyfluoroalkyl Chemicals in the U.S. Population: Data from the National Health and Nutrition Examination Survey (NHANES) 2003–2004 and Comparisons with NHANES 1999–2000. Environ. Health Perspect. 2007, 115 (11), 1596–1602. 10.1289/ehp.10598. 18007991 PMC2072821

[ref33] U.S. Environmental Protection Agency: PFAS structures in DSSTox. https://comptox.epa.gov/dashboard/chemical-lists/PFASSTRUCTV5 (accessed November 2, 2022).

[ref34] Harik S. I. Blood--brain barrier sodium/potassium pump: modulation by central noradrenergic innervation. Proc. Natl. Acad. Sci. U. S. A. 1986, 83 (11), 4067–4070. 10.1073/pnas.83.11.4067. 3012548 PMC323667

[ref35] Starnes H. M.; et al. A Critical Review and Meta-Analysis of Impacts of Per- and Polyfluorinated Substances on the Brain and Behavior. Frontiers in Toxicology 2022, 4, 881584 10.3389/ftox.2022.881584. 35480070 PMC9035516

[ref36] Trichopoulou A.; et al. Modified Mediterranean diet and survival: EPIC-elderly prospective cohort study. BMJ. 2005, 330 (7498), 991 10.1136/bmj.38415.644155.8F. 15820966 PMC557144

[ref37] Riboli E.; et al. European Prospective Investigation into Cancer and Nutrition (EPIC): study populations and data collection. Public Health Nutrition 2002, 5 (6b), 1113–1124. 10.1079/PHN2002394. 12639222

[ref38] Yang L.; et al. Neuroprotective Effects of the Triterpenoid, CDDO Methyl Amide, a Potent Inducer of Nrf2-Mediated Transcription. PLoS One 2009, 4 (6), e5757 10.1371/journal.pone.0005757. 19484125 PMC2684590

[ref39] Szakiel A.; et al. Fruit cuticular waxes as a source of biologically active triterpenoids. Phytochemistry Reviews 2012, 11 (2), 263–284. 10.1007/s11101-012-9241-9. 23519009 PMC3601259

[ref40] Ma Q. Role of Nrf2 in oxidative stress and toxicity. Annual Review of Pharmacology and Toxicology 2013, 53, 401–426. 10.1146/annurev-pharmtox-011112-140320. PMC4680839 23294312

[ref41] Jenner P. Oxidative stress in Parkinson’s disease. Ann. Neurol. 2003, 53 (S3), S26–S38. 10.1002/ana.10483. 12666096

[ref42] Ma C. M.; et al. The cytotoxic activity of ursolic acid derivatives. Eur. J. Med. Chem. 2005, 40 (6), 582–589. 10.1016/j.ejmech.2005.01.001. 15922841

[ref43] Krishnamurthy P.; et al. High-Throughput Screening and Characterization of a High-Density Soybean Mutant Library Elucidate the Biosynthesis Pathway of Triterpenoid Saponins. Plant and Cell Physiology 2019, 60 (5), 1082–1097. 10.1093/pcp/pcz025. 30753604

[ref44] Lee Y.-Y.; et al. Production, safety, health effects and applications of diacylglycerol functional oil in food systems: a review. Critical Reviews in Food Science and Nutrition 2020, 60 (15), 2509–2525. 10.1080/10408398.2019.1650001. 31418288

[ref45] Barbalace M. C.; et al. Antioxidant and Neuroprotective Activity of Extra Virgin Olive Oil Extracts Obtained from Quercetano Cultivar Trees Grown in Different Areas of the Tuscany Region (Italy). Antioxidants 2021, 10 (3), 421 10.3390/antiox10030421. 33801925 PMC8000409

[ref46] Jiang L.; et al. Serum level of brain-derived neurotrophic factor in Parkinson’s disease: a meta-analysis. Progress in Neuro-Psychopharmacology and Biological Psychiatry 2019, 88, 168–174. 10.1016/j.pnpbp.2018.07.010. 30017781

[ref47] Chmielarz P.; et al. Neurotrophic factors for disease-modifying treatments of Parkinson’s disease: gaps between basic science and clinical studies. Pharmacological Reports 2020, 72 (5), 1195–1217. 10.1007/s43440-020-00120-3. 32700249 PMC7550372

[ref48] Evatt M. L.; DeLong M. R.; Khazai N.; Rosen A.; Triche S.; Tangpricha V. Prevalence of Vitamin D Insufficiency in Patients With Parkinson Disease and Alzheimer Disease. Archives of Neurology 2008, 65 (10), 1348–1352. 10.1001/archneur.65.10.1348. 18852350 PMC2746037

[ref49] Bansal R.; et al. Exploring the potential of natural and synthetic neuroprotective steroids against neurodegenerative disorders: A literature review. Medicinal Research Reviews 2018, 38 (4), 1126–1158. 10.1002/med.21458. 28697282

[ref50] Hu H.; et al. The major cholesterol metabolite cholestane-3β,5α,6β-triol functions as an endogenous neuroprotectant. J. Neurosci. 2014, 34 (34), 11426–11438. 10.1523/JNEUROSCI.0344-14.2014. 25143622 PMC6615515

[ref51] Yan M.; et al. Characterization of a Synthetic Steroid 24-keto-cholest-5-en-3β, 19-diol as a Neuroprotectant. CNS Neuroscience & Therapeutics 2015, 21 (6), 486–495. 10.1111/cns.12378. 25678034 PMC6495820

