# Supplementary material for: Interpretable Machine Learning on Metabolomics Data Reveals Biomarkers for Parkinson’s Disease
Source: ACS Cent Sci. 2023 May 9;9(5):1035–45. doi: 10.1021/acscentsci.2c01468 (PMC10214508; doi:10.1021/acscentsci.2c01468)
Supplement: Supplementary file 1 — oc2c01468_si_001.pdf [file oc2c01468_si_001.pdf]

Supporting Information

Interpretable machine learning on metabolomics data reveals biomarkers for Parkinson's disease

J. Diana Zhang,[1,2] Chonghua Xue,[2] Vijaya B. Kolachalama,[2,3] and William A. Donald[1,*]

[1] *School of Chemistry, University of New South Wales, Sydney 2052, Australia*

[2] *Department of Medicine, Boston University School of Medicine, Boston, MA 02118, USA*

[3] *Department of Computer Science and Faculty of Computing & Data Sciences, Boston University, Boston, MA 02215, USA*

Correspondence to:

w.donald@unsw.edu.au

Table of Contents

Figure S1. .... S3

Figure S2. .... S4

Figure S3. .... S5

Table S1. .... S6

Table S2. .... S7

Table S3. .... S10

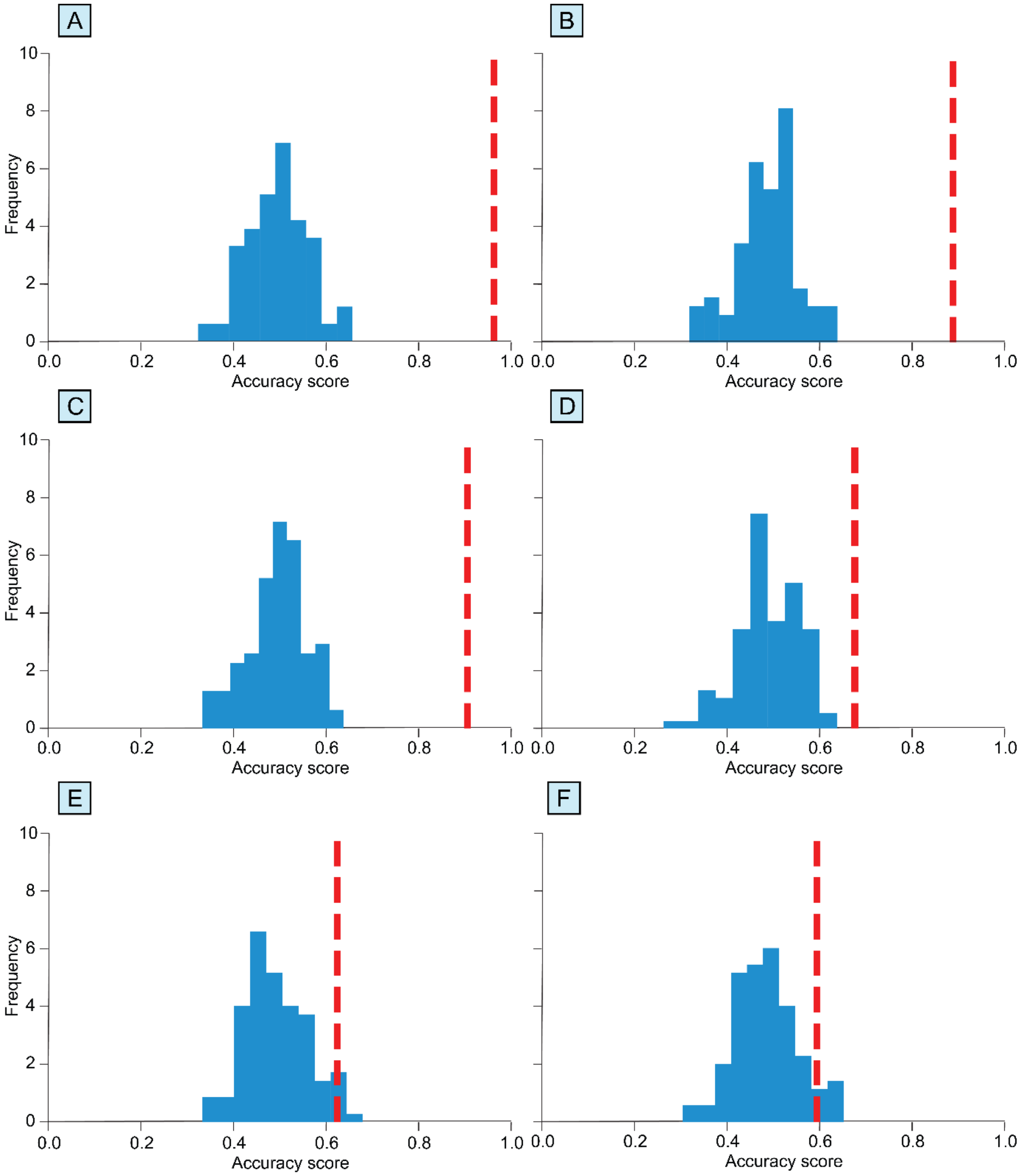

Figure S1. Randomly permuting the PD and healthy control data labels for the composite metabolomics dataset results in average accuracies (N = 100) that are statistically the same (1-sided t-test, 99.5% confidence interval) as 0.5 for all six machine learning models. An accuracy value of 0.5 corresponds to that obtained by 'random guessing' for binary classification. Histograms of accuracy scores (N = 100) obtained for permuted composite datasets using (A) neural network, (B) gradient boosting, (C) logistic regression, (D) random forest, (E) linear discriminant analysis, and (F) support vector machine models. The vertical, dashed red lines indicates the average accuracies obtained from the original datasets without permutation.

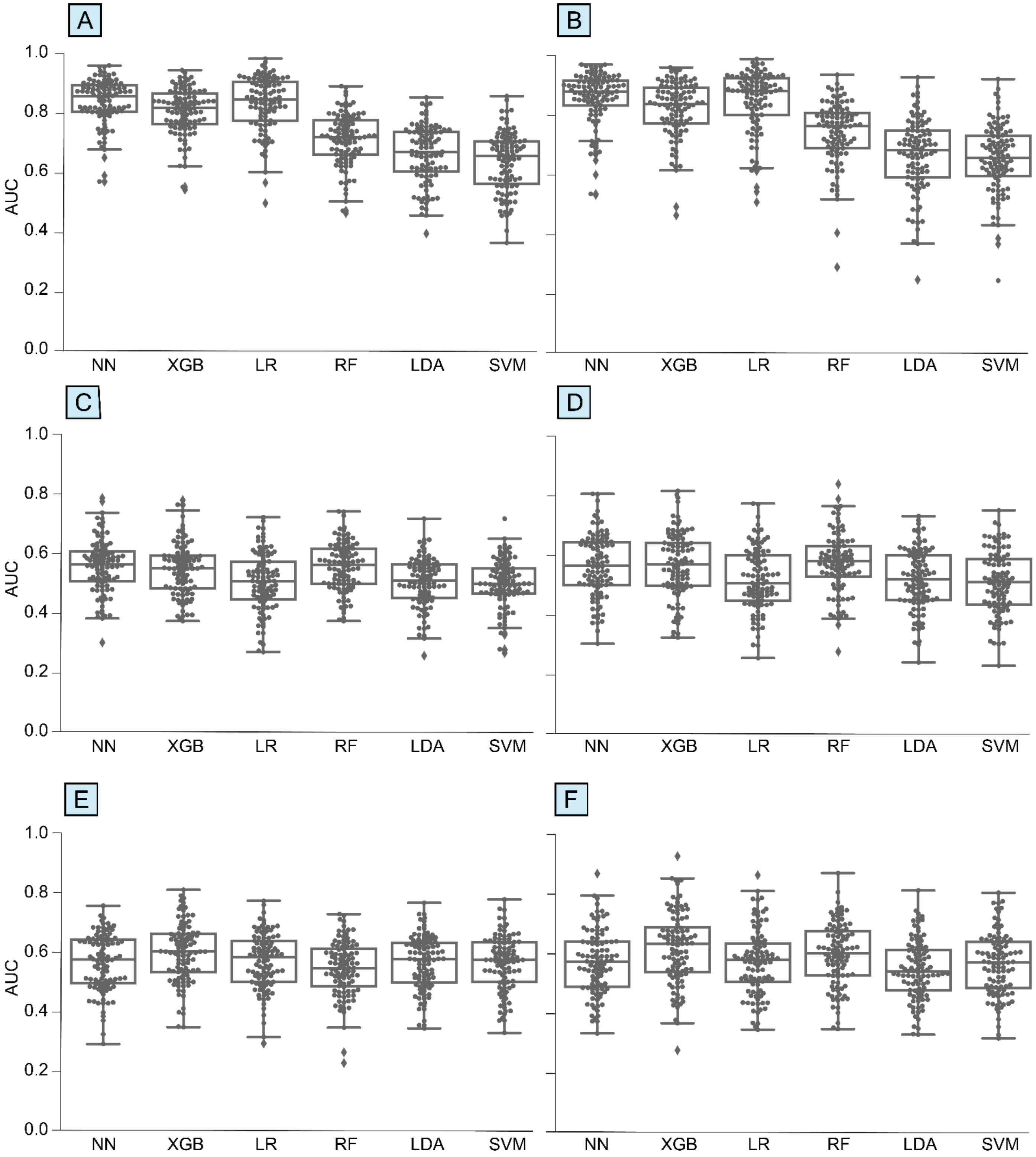

Figure S2. The performance for diagnosing PD using blood plasma metabolomics data depends strongly on the instrumental method that is used. Box-swarm plots of area under curves (AUC) of (A, C, E) receiver-operating curve (ROC) and (B, D, F) precision-recall (PR) for neural networks (NN), extreme gradient boosting (XGB), logistic regression (LR), random forest (RF), linear discriminant analysis (LDA), and support vector machine (SVM) classifiers for capillary electrophoresis-mass spectrometry (A, B), gas chromatography-mass spectrometry C, D), and liquid chromatography-mass spectrometry in negative mode (E, F).

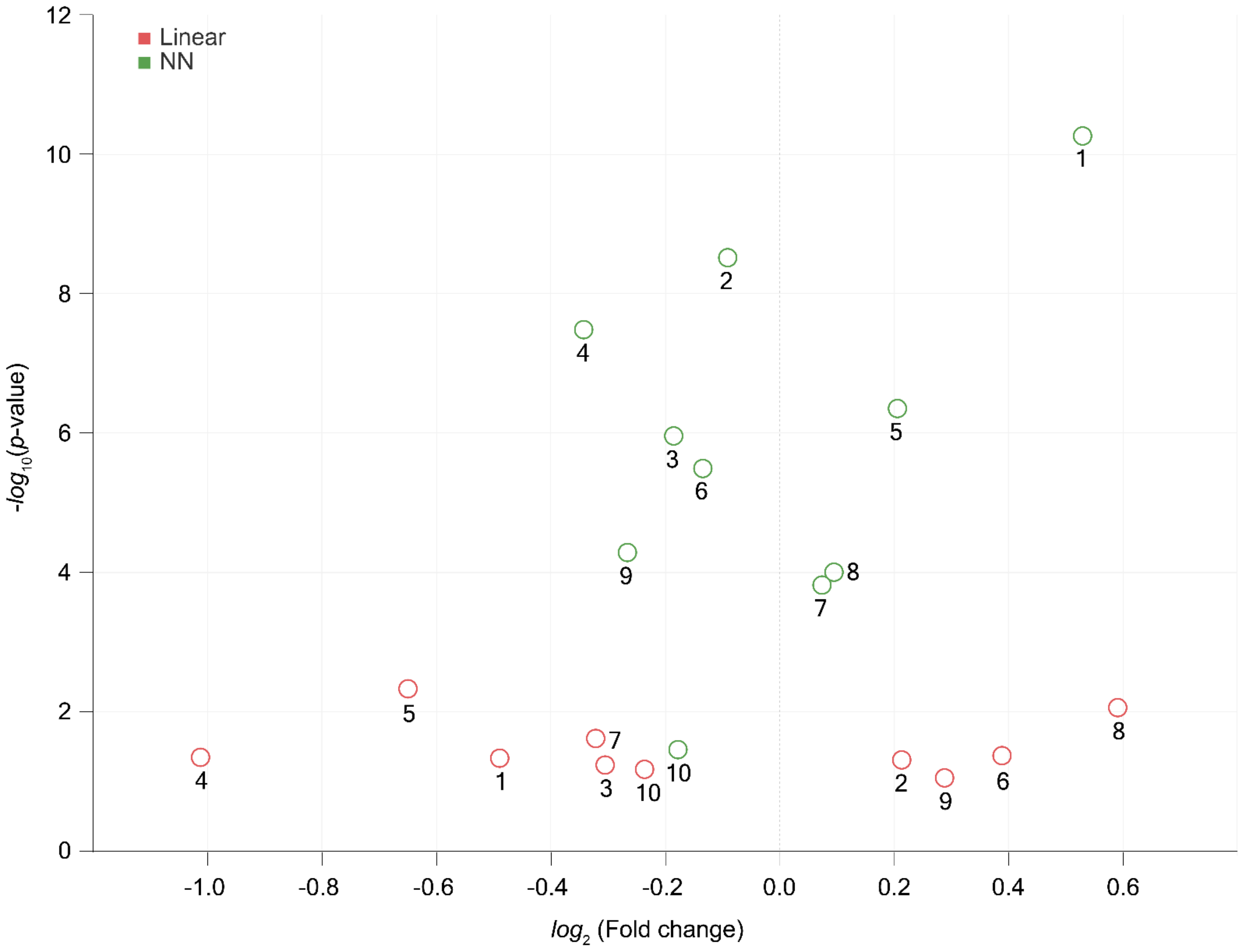

Figure S3. 'Volcano' plot of top scoring metabolite features from a linear model compared to a neural network model on the composite dataset reveals different model emphasis on fold change and *p*-value. The top 10 metabolite features selected from a linear-based model (red, Gonzalez-Riano *et al*.; Ref. 13 from main text) and those from CRANK-MS (green).

Table S1. Hyperparameter values applied to each algorithm based on GridSearch optimisation tuning for the composite dataset.

Method	Hyperparameters
Linear Discriminant Analysis	shrinkage: auto<br>solver: lsqr
Logistic Regression	C: 241<br>penalty: l1<br>solver: liblinear
Neural Network	batch_size: 16<br>lambda_l1: 0.0011697870951761014, lambda_l2: 0.0004719190005714674, num_epochs: 64
Random Forest	criterion: entropy<br>max_features: auto<br>min_samples_split: 5<br>n_estimators: 500
Support Vector Machine	C: 0.1<br>gamma: 0.0001<br>kernel: linear
Extreme Gradient Boosting	colsample_bytree: 0.4<br>learning_rate: 0.3<br>max_depth: 1<br>n_estimators: 50<br>reg_alpha: 0.5<br>reg_lambda: 0.05

Table S2. Summary of diagnostic performance obtained from each algorithmic framework and analytical method.

Analytical method	NN	RF	XGB	LR	SVM	LDA
GC-MS	Accuracy: 0.5197 ± 0.0855<br>Precision: 0.5147 ± 0.1313<br>Sensitivity/Recall: 0.5204 ± 0.1551<br>Specificity: 0.5416 ± 0.1520<br>F1 score: 0.4974 ± 0.1029<br>MCC: 0.0642 ± 0.1732<br>AUC (ROC): 0.5590 ± 0.0895<br>AUC (PR): 0.5661 ± 0.1054	Accuracy: 0.5123 ± 0.0763<br>Precision: 0.5183 ± 0.1548<br>Sensitivity/Recall: 0.4744 ± 0.1935<br>Specificity: 0.5845 ± 0.1678<br>F1 score: 0.4609 ± 0.1170<br>MCC: 0.0648 ± 0.1650<br>AUC (ROC): 0.5565 ± 0.0813<br>AUC (PR): 0.5750 ± 0.1008	Accuracy: 0.5177 ± 0.0791<br>Precision: 0.5107 ± 0.1210<br>Sensitivity/Recall: 0.4944 ± 0.1740<br>Specificity: 0.5676 ± 0.1404<br>F1 score: 0.4791 ± 0.1092<br>MCC: 0.0642 ± 0.1614<br>AUC (ROC): 0.5496 ± 0.0886<br>AUC (PR): 0.5676 ± 0.1092	Accuracy: 0.4863 ± 0.0775<br>Precision: 0.4691 ± 0.1149<br>Sensitivity/Recall: 0.4852 ± 0.1496<br>Specificity: 0.5022 ± 0.1231<br>F1 score: 0.4637 ± 0.1027<br>MCC: -0.0125 ± 0.1647<br>AUC (ROC): 0.5064 ± 0.0971<br>AUC (PR): 0.5189 ± 0.1056	Accuracy: 0.4813 ± 0.0712<br>Precision: 0.4699 ± 0.1124<br>Sensitivity/Recall: 0.4948 ± 0.1699<br>Specificity: 0.4957 ± 0.1472<br>F1 score: 0.4608 ± 0.1074<br>MCC: -0.0102 ± 0.1465<br>AUC (ROC): 0.4990 ± 0.0827<br>AUC (PR): 0.5103 ± 0.1059	Accuracy: 0.4850 ± 0.0737<br>Precision: 0.4756 ± 0.1150<br>Sensitivity/Recall: 0.4877 ± 0.1467<br>Specificity: 0.5047 ± 0.1433<br>F1 score: 0.4639 ± 0.0931<br>MCC: -0.0072 ± 0.1515<br>AUC (ROC): 0.5053 ± 0.0819<br>AUC (PR): 0.5189 ± 0.1022
CE-MS	Accuracy: 0.7425 ± 0.0829<br>Precision: 0.7556 ± 0.1343<br>Sensitivity/Recall: 0.7516 ± 0.1144<br>Specificity: 0.7478 ± 0.1465<br>F1 score: 0.7409 ± 0.0885<br>MCC: 0.5025 ± 0.1537<br>AUC (ROC): 0.8373 ± 0.0802<br>AUC (PR): 0.8571 ± 0.0905	Accuracy: 0.6216 ± 0.0947<br>Precision: 0.6569 ± 0.1558<br>Sensitivity/Recall: 0.6496 ± 0.1866<br>Specificity: 0.6390 ± 0.1942<br>F1 score: 0.6196 ± 0.1128<br>MCC: 0.3000 ± 0.1624<br>AUC (ROC): 0.7141 ± 0.0865<br>AUC (PR): 0.7411 ± 0.1061	Accuracy: 0.7184 ± 0.0813<br>Precision: 0.7275 ± 0.1247<br>Sensitivity/Recall: 0.7433 ± 0.1382<br>Specificity: 0.7109 ± 0.1506<br>F1 score: 0.7196 ± 0.0900<br>MCC: 0.4592 ± 0.1521<br>AUC (ROC): 0.8082 ± 0.0782<br>AUC (PR): 0.8207 ± 0.0946	Accuracy: 0.7478 ± 0.0911<br>Precision: 0.7529 ± 0.1318<br>Sensitivity/Recall: 0.7581 ± 0.1157<br>Specificity: 0.7456 ± 0.1382<br>F1 score: 0.7462 ± 0.0990<br>MCC: 0.5048 ± 0.1793<br>AUC (ROC): 0.8325 ± 0.0914<br>AUC (PR): 0.8464 ± 0.1044	Accuracy: 0.5947 ± 0.0864<br>Precision: 0.6030 ± 0.1276<br>Sensitivity/Recall: 0.5939 ± 0.1544<br>Specificity: 0.6085 ± 0.1284<br>F1 score: 0.5845 ± 0.1139<br>MCC: 0.2048 ± 0.1747<br>AUC (ROC): 0.6386 ± 0.1002<br>AUC (PR): 0.6536 ± 0.1161	Accuracy: 0.6153 ± 0.0839<br>Precision: 0.6257 ± 0.1306<br>Sensitivity/Recall: 0.6261 ± 0.1342<br>Specificity: 0.6191 ± 0.1359<br>F1 score: 0.6126 ± 0.1002<br>MCC: 0.2479 ± 0.1677<br>AUC (ROC): 0.6641 ± 0.0968<br>AUC (PR): 0.6672 ± 0.1234
LC-MS (+)	Accuracy: 0.9463 ± 0.0410<br>Precision: 0.9447 ± 0.0580<br>Sensitivity/Recall: 0.9507 ± 0.0613<br>Specificity: 0.9440 ± 0.0621<br>F1 score: 0.9455 ± 0.0415	Accuracy: 0.7725 ± 0.1100<br>Precision: 0.7958 ± 0.1534<br>Sensitivity/Recall: 0.8016 ± 0.1582<br>Specificity: 0.7807 ± 0.1821<br>F1 score: 0.7748 ± 0.1095	Accuracy: 0.9000 ± 0.0603<br>Precision: 0.8960 ± 0.0818<br>Sensitivity/Recall: 0.9112 ± 0.0817<br>Specificity: 0.8953 ± 0.0791<br>F1 score: 0.8997 ± 0.0608	Accuracy: 0.9341 ± 0.0393<br>Precision: 0.9370 ± 0.0512<br>Sensitivity/Recall: 0.9329 ± 0.0676<br>Specificity: 0.9355 ± 0.0524<br>F1 score: 0.9327 ± 0.0411	Accuracy: 0.7847 ± 0.0864<br>Precision: 0.8095 ± 0.1204<br>Sensitivity/Recall: 0.7641 ± 0.1233<br>Specificity: 0.8177 ± 0.1185<br>F1 score: 0.7764 ± 0.0931	Accuracy: 0.8069 ± 0.0781<br>Precision: 0.8293 ± 0.1120<br>Sensitivity/Recall: 0.7891 ± 0.1158<br>Specificity: 0.8368 ± 0.1090<br>F1 score: 0.8000 ± 0.0827

Analytical method	NN	RF	XGB	LR	SVM	LDA
	MCC:<br>0.8939 ± 0.0809<br>AUC (ROC):<br>0.9825 ± 0.0215<br>AUC (PR):<br>0.9836 ± 0.0217	MCC:<br>0.5885 ± 0.1768<br>AUC (ROC):<br>0.8944 ± 0.0794<br>AUC (PR):<br>0.9114 ± 0.0710	MCC:<br>0.8047 ± 0.1166<br>AUC (ROC):<br>0.9681 ± 0.0291<br>AUC (PR):<br>0.9661 ± 0.0360	MCC:<br>0.8691 ± 0.0780<br>AUC (ROC):<br>0.9722 ± 0.0286<br>AUC (PR):<br>0.9759 ± 0.0263	MCC:<br>0.5823 ± 0.1672<br>AUC (ROC):<br>0.8561 ± 0.0728<br>AUC (PR):<br>0.8687 ± 0.0779	MCC:<br>0.6258 ± 0.1504<br>AUC (ROC):<br>0.8778 ± 0.0658<br>AUC (PR):<br>0.8767 ± 0.0858
LC-MS (-)	Accuracy:<br>0.5447 ± 0.0859<br>Precision:<br>0.5421 ± 0.1241<br>Sensitivity/Recall:<br>0.5775 ± 0.1548<br>Specificity:<br>0.5338 ± 0.1509<br>F1 score:<br>0.5428 ± 0.0984<br>MCC:<br>0.1136 ± 0.1778<br>AUC (ROC):<br>0.5653 ± 0.0947<br>AUC (PR):<br>0.5728 ± 0.1068	Accuracy:<br>0.5037 ± 0.0912<br>Precision:<br>0.5279 ± 0.1423<br>Sensitivity/Recall:<br>0.5506 ± 0.1920<br>Specificity:<br>0.5105 ± 0.2227<br>F1 score:<br>0.5022 ± 0.1046<br>MCC:<br>0.0645 ± 0.1663<br>AUC (ROC):<br>0.5453 ± 0.0923<br>AUC (PR):<br>0.5966 ± 0.1055	Accuracy:<br>0.5563 ± 0.0948<br>Precision:<br>0.5625 ± 0.1329<br>Sensitivity/Recall:<br>0.5794 ± 0.1617<br>Specificity:<br>0.5625 ± 0.1713<br>F1 score:<br>0.5498 ± 0.1016<br>MCC:<br>0.1452 ± 0.1827<br>AUC (ROC):<br>0.5979 ± 0.0972<br>AUC (PR):<br>0.6193 ± 0.1206	Accuracy:<br>0.5540 ± 0.0927<br>Precision:<br>0.5476 ± 0.1224<br>Sensitivity/Recall:<br>0.5865 ± 0.1494<br>Specificity:<br>0.5375 ± 0.1511<br>F1 score:<br>0.5530 ± 0.1011<br>MCC:<br>0.1251 ± 0.1877<br>AUC (ROC):<br>0.5688 ± 0.0930<br>AUC (PR):<br>0.5729 ± 0.1095	Accuracy:<br>0.5433 ± 0.0909<br>Precision:<br>0.5382 ± 0.1222<br>Sensitivity/Recall:<br>0.5713 ± 0.1497<br>Specificity:<br>0.5325 ± 0.1458<br>F1 score:<br>0.5400 ± 0.1047<br>MCC:<br>0.1052 ± 0.1814<br>AUC (ROC):<br>0.5620 ± 0.0933<br>AUC (PR):<br>0.5652 ± 0.1038	Accuracy:<br>0.5423 ± 0.0904<br>Precision:<br>0.5347 ± 0.1219<br>Sensitivity/Recall:<br>0.5787 ± 0.1572<br>Specificity:<br>0.5232 ± 0.1417<br>F1 score:<br>0.5413 ± 0.1108<br>MCC:<br>0.1031 ± 0.1784<br>AUC (ROC):<br>0.5637 ± 0.0907<br>AUC (PR):<br>0.5477 ± 0.0990
LC-MS drug-naïve PD *vs* control	Accuracy:<br>0.7669 ± 0.0488<br>Precision:<br>0.8149 ± 0.0772<br>Sensitivity/Recall:<br>0.7952 ± 0.0779<br>Specificity:<br>0.7348 ± 0.1145<br>F1 score:<br>0.7996 ± 0.0463<br>MCC:<br>0.5296 ± 0.0983<br>AUC (ROC):<br>0.8426 ± 0.0453<br>AUC (PR):<br>0.8958 ± 0.0460	Accuracy:<br>0.7138 ± 0.0654<br>Precision:<br>0.7390 ± 0.0932<br>Sensitivity/Recall:<br>0.8228 ± 0.0878<br>Specificity:<br>0.5749 ± 0.1560<br>F1 score:<br>0.7714 ± 0.0528<br>MCC:<br>0.4156 ± 0.1328<br>AUC (ROC):<br>0.8112 ± 0.0634<br>AUC (PR):<br>0.8768 ± 0.0486	Accuracy:<br>0.7471 ± 0.0609<br>Precision:<br>0.7805 ± 0.0853<br>Sensitivity/Recall:<br>0.8088 ± 0.0777<br>Specificity:<br>0.6700 ± 0.1273<br>F1 score:<br>0.7893 ± 0.0540<br>MCC:<br>0.4841 ± 0.1219<br>AUC (ROC):<br>0.8324 ± 0.0599<br>AUC (PR):<br>0.8866 ± 0.0559	Accuracy:<br>0.7584 ± 0.0515<br>Precision:<br>0.8231 ± 0.0782<br>Sensitivity/Recall:<br>0.7624 ± 0.0802<br>Specificity:<br>0.7613 ± 0.1080<br>F1 score:<br>0.7865 ± 0.0504<br>MCC:<br>0.5189 ± 0.1009<br>AUC (ROC):<br>0.8260 ± 0.0526<br>AUC (PR):<br>0.8714 ± 0.0597	Accuracy:<br>0.7640 ± 0.0521<br>Precision:<br>0.8247 ± 0.0705<br>Sensitivity/Recall:<br>0.7697 ± 0.0807<br>Specificity:<br>0.7612 ± 0.0993<br>F1 score:<br>0.7920 ± 0.0525<br>MCC:<br>0.5263 ± 0.1029<br>AUC (ROC):<br>0.8269 ± 0.0528<br>AUC (PR):<br>0.8821 ± 0.0491	Accuracy:<br>0.7551 ± 0.0502<br>Precision:<br>0.8268 ± 0.0715<br>Sensitivity/Recall:<br>0.7470 ± 0.0770<br>Specificity:<br>0.7722 ± 0.0957<br>F1 score:<br>0.7808 ± 0.0513<br>MCC:<br>0.5126 ± 0.1002<br>AUC (ROC):<br>0.7804 ± 0.0598<br>AUC (PR):<br>0.7983 ± 0.0679
LC-MS medicated PD *vs* control	Accuracy:<br>0.7897 ± 0.0436<br>Precision:<br>0.8317 ± 0.0525<br>Sensitivity/Recall:<br>0.8851 ± 0.0546	Accuracy:<br>0.7342 ± 0.0445<br>Precision:<br>0.7527 ± 0.0531<br>Sensitivity/Recall:<br>0.9356 ± 0.0447	Accuracy:<br>0.7941 ± 0.0429<br>Precision:<br>0.8119 ± 0.0521<br>Sensitivity/Recall:<br>0.9262 ± 0.0394	Accuracy:<br>0.7787 ± 0.0425<br>Precision:<br>0.8297 ± 0.0497<br>Sensitivity/Recall:<br>0.8673 ± 0.0503	Accuracy:<br>0.7655 ± 0.0434<br>Precision:<br>0.8244 ± 0.0495<br>Sensitivity/Recall:<br>0.8529 ± 0.0580	Accuracy:<br>0.7668 ± 0.0427<br>Precision:<br>0.8289 ± 0.0517<br>Sensitivity/Recall:<br>0.8488 ± 0.0542

Analytical method	NN	RF	XGB	LR	SVM	LDA
	Specificity: 0.5679 ± 0.1252<br>F1 score: 0.8554 ± 0.0323<br>MCC: 0.4794 ± 0.1041<br>AUC (ROC): 0.8217 ± 0.0499<br>AUC (PR): 0.8963 ± 0.0480	Specificity: 0.2605 ± 0.1176<br>F1 score: 0.8322 ± 0.0296<br>MCC: 0.2737 ± 0.1210<br>AUC (ROC): 0.7671 ± 0.0570<br>AUC (PR): 0.8884 ± 0.0427	Specificity: 0.4856 ± 0.1181<br>F1 score: 0.8637 ± 0.0295<br>MCC: 0.4724 ± 0.1087<br>AUC (ROC): 0.8406 ± 0.0537<br>AUC (PR): 0.9230 ± 0.0353	Specificity: 0.5732 ± 0.1067<br>F1 score: 0.8463 ± 0.0320<br>MCC: 0.4567 ± 0.0988<br>AUC (ROC): 0.8095 ± 0.0512<br>AUC (PR): 0.8908 ± 0.0473	Specificity: 0.5633 ± 0.1061<br>F1 score: 0.8362 ± 0.0335<br>MCC: 0.4286 ± 0.0968<br>AUC (ROC): 0.8051 ± 0.0471<br>AUC (PR): 0.8977 ± 0.0378	Specificity: 0.5780 ± 0.1145<br>F1 score: 0.8366 ± 0.0321<br>MCC: 0.4345 ± 0.1002<br>AUC (ROC): 0.7387 ± 0.0575<br>AUC (PR): 0.8305 ± 0.0504
Composite dataset	Accuracy: 0.9576 ± 0.0447<br>Precision: 0.9618 ± 0.0613<br>Sensitivity/Recall: 0.9533 ± 0.0708<br>Specificity: 0.9646 ± 0.0585<br>F1 score: 0.9550 ± 0.0491<br>MCC: 0.9176 ± 0.0863<br>AUC (ROC): 0.9939 ± 0.0180<br>AUC (PR): 0.9950 ± 0.0144	Accuracy: 0.6772 ± 0.1238<br>Precision: 0.7278 ± 0.1840<br>Sensitivity/Recall: 0.6889 ± 0.2327<br>Specificity: 0.7229 ± 0.2167<br>F1 score: 0.6562 ± 0.1496<br>MCC: 0.4330 ± 0.1920<br>AUC (ROC): 0.8290 ± 0.0992<br>AUC (PR): 0.8356 ± 0.0994	Accuracy: 0.8883 ± 0.0656<br>Precision: 0.8968 ± 0.0946<br>Sensitivity/Recall: 0.8826 ± 0.1084<br>Specificity: 0.9061 ± 0.0879<br>F1 score: 0.8820 ± 0.0701<br>MCC: 0.7872 ± 0.1191<br>AUC (ROC): 0.9699 ± 0.0278<br>AUC (PR): 0.9683 ± 0.0308	Accuracy: 0.9059 ± 0.0681<br>Precision: 0.9078 ± 0.0928<br>Sensitivity/Recall: 0.8983 ± 0.0853<br>Specificity: 0.9166 ± 0.0898<br>F1 score: 0.8995 ± 0.0723<br>MCC: 0.8149 ± 0.1317<br>AUC (ROC): 0.9684 ± 0.0367<br>AUC (PR): 0.9689 ± 0.0369	Accuracy: 0.5952 ± 0.0798<br>Precision: 0.5915 ± 0.1182<br>Sensitivity/Recall: 0.6020 ± 0.1434<br>Specificity: 0.6103 ± 0.1223<br>F1 score: 0.5813 ± 0.0939<br>MCC: 0.2133 ± 0.1546<br>AUC (ROC): 0.6471 ± 0.0931<br>AUC (PR): 0.6606 ± 0.1108	Accuracy: 0.6255 ± 0.0779<br>Precision: 0.6215 ± 0.1185<br>Sensitivity/Recall: 0.6337 ± 0.1451<br>Specificity: 0.6372 ± 0.1244<br>F1 score: 0.6122 ± 0.0938<br>MCC: 0.2724 ± 0.1517<br>AUC (ROC): 0.6813 ± 0.0909<br>AUC (PR): 0.6340 ± 0.1194

Table S3. Potential alternative isomers of Dammarenediol II and Vitamin D2.

m/z	Class	Putative annotation	Disposition in blood	Monoisotopic mass
467.3822	Triterpenoid	Dammarenediol II	Detected in blood but not quantified	444.3967
		4,4-Dimethyl-14alpha-hydroxymethyl-5alpha-cholesta-8-en-3beta-ol	Expected but not quantified	
		gamma-Taraxastane-3,20-diol	Expected but not quantified	
		Gladiatoside B3	Expected but not quantified	
		3beta-Cycloartane-3,25-diol	Expected but not quantified	
		3beta-Cycloartane-3,29-diol	Expected but not quantified	
		Arabidiol	Expected but not quantified	
379.3289	Vitamin D steroids	Vitamin D2	Detected in blood and quantified	396.3392
		Isotachysterol	Detected in blood but not quantified	
	Cholestane steroids	4a-Methyl-5a-cholesta-8,24-dien-3-one	Expected but not quantified	
		3-Keto-4-methylzymosterol	Expected but not quantified	
		4-Methylzymosterol intermediate 2	Expected but not quantified	
		31-Nordehydrolanosterol	Expected but not quantified	
		4alpha-methyl-5alpha-cholesta-8,14,24-trien-3beta-ol	Expected but not quantified	
	Ergostane steroids	5-Dehydroepisterol	Expected but not quantified	
		5,7,24(28)-Ergostatrienol	Expected but not quantified	
		Ergosterol	Detected in blood and quantified	
		(3S,9S,10R,13R,17R)-17-((2R,5R,E)-5,6-Dimethylhept-3-en-2-yl)-10,13-dimethyl-2,3,4,9,10,11,12,13,14,15,16,17-dodecahydro-1H-cyclopenta[a]phenanthren-3-ol	Detected in blood but not quantified	
		Episterone	Expected but not quantified	
	Steroid	24-Methylenepollinastanone	Expected but not quantified	
